# Postoperative Atrial Fibrillation Following Off-Pump Coronary Artery Bypass Graft Surgery: Elderly Versus Young Patients

**DOI:** 10.7759/cureus.39232

**Published:** 2023-05-19

**Authors:** Ashok Kumar, Redoy Ranjan, Asit B Adhikary

**Affiliations:** 1 Department of Cardiac Surgery, Bangabandhu Sheikh Mujib Medical University, Dhaka, BGD; 2 Department of Surgical Sciences, The University of Edinburgh, Edinburgh, GBR; 3 Department of Biological Sciences, Royal Holloway, University of London, London, GBR

**Keywords:** duration of hospital stay, off-pump coronary artery bypass graft surgery, young, elderly, postoperative atrial fibrillation

## Abstract

Background

Atrial fibrillation (AF) is one of the common rhythm disturbances that occur after coronary artery bypass graft (CABG) surgery. Postoperative atrial fibrillation (POAF) can lead to thromboembolic events, hemodynamic instability, and prolonged hospital stay, affecting morbidity and influencing short and long-term outcomes after CABG.

Methodology

This prospective comparative study was conducted between May 2018 and April 2020. This study aimed to compare the prevalence of POAF following off-pump coronary artery bypass graft surgery (OPCAB) between elderly and young patients. Additionally, we aimed to determine the risk factors associated with POAF following OPCAB in the elderly compared to young patients. Patients aged ≥65 years were considered elderly, and those aged <65 years were considered young. A total of 120 patients (60 in the elderly group and 60 in the young group) were included in this study and evaluated to correlate the preoperative and intraoperative risk factors with postoperative outcomes during the hospital stay.

Results

The prevalence of POAF following OPCAB in the elderly was significantly higher compared to young patients (48.3% vs. 20%,p = 0.002). The elderly group also had a significantly longer intensive care unit stay (p = 0.001) and hospital stay (p = 0.001). In an unadjusted logistic regression model, age (odds ratio (OR) = 3.74, 95% confidence interval (CI) = 1.66-8.41, p = 0.001), preoperative plasma B-type natriuretic peptide (OR = 1.01, 95% CI = 1.00-1.01, p = 0.001), and left atrial diameter (OR = 1.10, 95% CI = 1.03-1.17, p = 0.001) were significantly associated with POAF. However, in an adjusted logistic regression model, age was found to be an independent predictor (OR = 1.31, 95% CI = 1.14-1.52, p < 0.0001) of POAF following OPCAB. Although stroke developed in the elderly (p >0.05), no mortality was observed postoperatively.

Conclusions

The prevalence of POAF following OPCAB in the elderly is higher than in young patients. Advancing age is an independent predictor of POAF following OPCAB.

## Introduction

Atrial fibrillation (AF) is a disorder of cardiac rhythm accounting for about one-third of arrhythmia cases and presents a difficult clinical challenge. Approximately 6% of people older than 65 years and 1% of the general population has a history of AF [1]. The incidence of AF in Asia is 5.38/1,000 persons per year [2]. There are two major theories concerning the mechanism of AF, namely, (i) re-entry involving one or more circuits, and (ii) enhanced automaticity in one or several rapidly depolarizing foci [3]. Off-pump coronary artery bypass graft surgery (OPCAB) is safe and associated with lower postoperative myocardial infarction (MI) incidence, perioperative complications, and hospital mortality than conventional or on-pump coronary artery bypass graft (CABG) [4]. Rhythm disturbances in the postoperative period depend on electrolyte shifts associated with revascularization, temporary ischemia, perioperative trauma, epicardial inflammatory reaction, and transient increase of sympathetic activity [5]. Postoperative atrial fibrillation (POAF) occurs in 20% to 40% of patients after CABG [1]. Dieberg et al. reported that the incidence of POAF is 19.4% in OPCAB [6]. In a meta-analysis, Athanasiou et al. reported that the incidence of POAF is 22% in elderly patients undergoing OPCAB [7].

POAF usually occurs within the first three days after the operation, although it may occur later. More than 90% of patients have no prior history of atrial arrhythmia and are in normal sinus rhythm within six to eight weeks after the operation [1]. Advancing age is associated with degenerative changes in the atrial myocardium, which causes a significant increase in myocardial thickness due to increased collagen content, fibrosis, and deposition of cardiac amyloid and lipofuscin [8,9]. In addition, elastic and collagenous tissue increases in the conductive system, and fat accumulates around the sinoatrial (SA) node leading to a marked decline in the total amount of pacemaker cells in the SA node after the age of 60 years, which might increase the susceptibility of the elderly to develop POAF [9].

Xu et al. reported that abnormal left atrial (LA) diameter and lower left ventricular ejection fraction (LVEF) are closely related to myocardial fibrosis and structure reconstruction in which surgical trauma, postoperative inflammation, and oxidative stress may trigger POAF after CABG. In addition, smoking, low-density lipoprotein, and statins are significantly related to POAF after OPCAB [10]. Kouchoukos et al. reported that systemic arterial hypertension, decreased left ventricular function, chronic obstructive pulmonary disease (COPD), chronic renal failure, diabetes, and withdrawal of β-blockers are responsible for POAF after CABG [1]. Preoperative higher plasma B-type natriuretic peptide (BNP) level independently predicts POAF after isolated CABG [11,12]. Preoperative HbA1c independently predicts the occurrence of AF after isolated OPCAB [13]. Newby et al. observed that AF is also associated with hyperthyroidism and alcohol consumption [14]. Furthermore, the independent risk factor of POAF includes the male gender, and the incidence is lower in women following isolated CABG [8,15].

POAF is associated with the number of coronary arteries bypassed, the location of coronary anastomosis (diagonal or posterior descending artery), and net fluid balance on the day of operation [16]. Nonetheless, operative trauma, local inflammation, elevations in atrial pressure from postoperative ventricular stunning, chemical stimulation from catecholamines and other inotropic agents, reflex sympathetic activation from volume loss, parasympathetic activation, anemia or pain, fever from atelectasis or infection, and hypoglycemia during the operation may predispose to POAF [17]. In addition, transient regional ischemia of the myocardium due to surgical methods applied in OPCAB shows the influence of the technique on the incidence of POAF [5]. Therefore, this study aimed to observe the prevalence and risk factors of POAF following OPCAB compared to elderly and young patients.

## Materials and methods

This prospective comparative study was conducted in the Department of Cardiac Surgery, Bangabandhu Sheikh Mujib Medical University, Dhaka, Bangladesh, from May 2018 to April 2020. According to the definition of elderly by Geriatrics and Gerontology International, patients aged 65 years and above were considered elderly [18]. Patients aged <65 years were considered young. The purposive sampling method was followed as the sampling technique. The study population (n = 120) was grouped into the elderly group (n = 60) and the young group (n = 60).

Data collection was done through face-to-face interviews and a semi-structured questionnaire. Inclusion criteria were all admitted patients scheduled for elective isolated OPCAB. Exclusion criteria included pre-existing AF and other arrhythmias, taking antiarrhythmic drugs except for β-blockers, pre-existing thyroid disorders, congestive cardiac failure, other concomitant cardiac procedures, acute coronary syndrome within two months, emergency OPCAB, hepatic impairment, chronic kidney disease stage 3b-5, previous stroke, significant carotid artery disease, and intraoperative conversion to on-pump CABG. We utilized both primary and secondary data sources for this study. The primary sources were gathered via face-to-face interviews and semi-structured questionnaires, and the secondary sources were acquired through clinical observations and laboratory workup.

Surgical technique, POAF monitoring, and rhythm control

All patients were approached through standard median sternotomy under the standard anesthetic protocol. Activated clotting time (ACT) was maintained at >350 seconds just before completing the left internal mammary artery harvest. Proximal anastomoses were done using a side-biting clamp following the parachute technique. Target coronary arteries were stabilized using the tissue-stabilizing system, and appropriate-sized intracoronary shunts were used in all cases to maintain distal perfusion and achieve a bloodless operative field. All patients were taken to the intensive care unit (ICU) immediately after completion of the surgery and with the continuation of mechanical ventilation. Mechanical ventilation was continued until the patient fulfilled the standard extubation criteria.

In the ICU, all patients were monitored for heart rate and rhythm by continuous electrocardiography (ECG) monitoring. In addition, a 12-lead ECG was performed whenever POAF was noticed by continuous monitoring. All patients received inotrope support and other medications per hospital protocol. β-blockers were reinstituted immediately after operation for individuals who were on β-blockers preoperatively. Amiodarone was used for rhythm control of POAF following standard guidelines. Additionally, a low-dose β-blocker was used to maintain sinus rhythm when necessary. Serum electrolyte (Na^+^, K^+^, Ca^++^, and Mg^++^) levels were routinely checked in the early postoperative period. When any imbalance was noticed, the levels were corrected following the standard protocol. Subsequently, the patients were transferred to the post-ICU and then to the postoperative ward whenever appropriate, according to the consultant’s judgment. After meeting the discharge criteria, the patients were discharged from the hospital.

Data analysis

Statistical analysis was conducted using SPSS Statistics for Windows version 25.0 (IBM Corp., Armonk, NY, USA). Comparisons between groups were established using unpaired Student’s t-test, Fisher’s exact test, and chi-square test. Finally, logistic regression analysis was used to control confounding variables to neutralize their effect on the outcome variable. The numerical data were expressed as mean and standard deviation. The numbers and percentages were used to express the categorical data. Observations were recorded as statistically significant if p-values <0.05.

Ethical considerations

Ethical clearance for this study was obtained by the Institutional Review Board, Bangabandhu Sheikh Mujib Medical University, Shahbagh, Dhaka, Bangladesh (approval number: BSMMU/2018/5539). Informed written consent was obtained from all the respondents involved in this study. A quality assurance system was applied to ensure the validity of the observations and findings. The reliability of the data was confirmed to validate that it was derived accurately from raw data. Quality control measures were applied in every stage of data handling to ensure consistency and appropriate processing of data.

## Results

A total of 120 patients (elderly group: 60 patients and young group: 60 patients) underwent elective isolated OPCAB. All patients were evaluated for preoperative clinical data, intraoperative risk factors, and postoperative outcomes. Table 1 compares preoperative demographic, anthropometric, clinical, biochemical, radiological, echocardiographic, and angiographic variables. The distribution of gender, hypertension, history of old MI, plasma BNP, LA diameter, left ventricular internal diameter at end-diastole (LVIDD), LVEF, and left main coronary artery involvement was statistically significant (p < 0.05).

**Table 1:** Preoperative demographic, anthropometric, clinical, biochemical, radiological, echocardiographic, and angiographic characteristics. BMI: body mass index; MI: myocardial infarction; COPD: chronic obstructive pulmonary disease; BNP: B-type natriuretic peptide; CTR: cardiothoracic ratio; LA: left atrial; LVIDD: left ventricular internal diameter at end-diastole; LVEF: left ventricular ejection fraction; DVD: double vessel disease; TVD: triple vessel disease: RCA: right coronary artery

Variables	Elderly (n = 60) f (%)	Young (n = 60) f (%)	P-value
Sex			
Male	52 (86.6%)	38 (63.3%)	0.006
Female	08 (13.3%)	22 (36.7%)	
BMI (kg/m^2^) (mean ± SD)	25.59 ± 3.01	25.02 ± 3.20	0.32
Shortness of breath	11 (18.3%)	9 (15%)	0.80
Chest pain	55 (91.7%)	56 (93.3%)	1.0
Smoking	27 (45%)	20 (33%)	0.26
Alcohol consumption	03 (5%)	04 (6.7%)	1.0
Hypertension	49 (81.7%)	38 (63.3%)	0.040
History of old MI	55 (91.7%)	44 (73.3%)	0.015
Diabetes mellitus	28 (46.7%)	22 (36.7%)	0.35
Dyslipidemia	46 (76.6%)	49 (81.6%)	0.50
COPD	05 (8.3%)	03 (5%)	0.71
Serum creatinine (mg/dL) (Mean±SD)	1.29 ± 0.23	1.22 ± 0.22	0.07
Plasma BNP (pg/ml) (mean ± SD)	150.24 ± 81.34	120.10 ± 54.69	0.019
Higher CTR	18 (30%)	10 (16.7%)	0.13
LA diameter (mm) (mean ± SD)	43.61 ± 6.48	40.03 ± 5.64	0.002
LVIDD (mm) (mean ± SD)	59.91 ± 6.59	56.76 ± 6.88	0.012
LVEF (%) (mean ± SD)	41.42 ± 7.29	45.88 ± 7.96	0.002
Number of diseased vessels (mean ± SD)	2.77 ± 0.42	2.68 ± 0.46	0.31
DVD	14 (23.3%)	19 (31.7%)	0.41
TVD	46 (76.7%)	41 (68.3%)	0.41
Left main stem involvement	27 (45%)	15 (25%)	0.035
RCA total/near-total occlusion	26 (43.3%)	22 (36.7%)	0.57
In-stent restenosis	10 (16.7%)	06 (10%)	0.42

Table 2 shows the distribution of different intraoperative variables between the groups. Among the study population, coronary endarterectomy was statistically significant (p < 0.05).

**Table 2:** The distribution of intraoperative characteristics. Note: Coronary endarterectomy (n = 20) in the elderly group and (n = 9) in the young group.

Variables	Elderly (n = 60) f (%)	Young (n = 60) f (%)	Elderly (n = 20) f (%)	Young (n = 9) f (%)	P-value
Duration of surgery (hours) (mean ± SD)	4.79 ± 0.65	4.59 ± 0.64			0.09
Number of grafts (mean ± SD)	3.20 ± 0.73	3.03 ± 0.73			0.21
Coronary endarterectomy	20 (33.3%)	09 (15%)			0.032
The territory of coronary endarterectomy					
Left anterior descending artery			09 (45%)	03 (33.3%)	
Obtuse marginal artery			01 (5%)	01 (11.1%)	
Diagonal artery			05 (25%)	02 (22.2%)	
Right coronary artery			03 (15%)	02 (22.2%)	
Posterior descending artery			02 (10%)	01 (11.1%)	
In double vessel			02 (10%)	01 (11.1%)	

The distribution of postoperative serum electrolyte levels is shown in Table 3.

**Table 3:** Postoperative serum electrolyte levels. POD: postoperative day

Postoperative serum electrolytes (mmol/L)	Elderly (n = 60) (mean ± SD)	Young (n = 60) (mean ± SD)	P-value
Serum sodium (Na^+^) level (mmol/L)			
POD 0	138.02 ± 3.63	137.42 ± 3.73	0.37
POD 1	136.80 ± 3.23	136.76 ± 3.09	0.94
POD 2	136.26 ± 3.42	136.56 ± 3.0	0.61
POD 3	136.06 ± 2.98	136.02 ± 1.87	0.93
Serum potassium (K^+^ ) level (mmol/L)			
POD 0	4.05 ± 0.38	4.07 ± 0.20	0.72
POD 1	4.06 ± 0.42	4.04 ± 0.20	0.82
POD 2	4.04 ± 0.36	4.02 ± 0.29	0.61
POD 3	4.03 ± 0.20	4.02 ± 0.16	0.62
Serum calcium (Ca^++^) level (mmol/L)			
POD 0	1.16 ± 0.03	1.15 ± 0.04	0.13
POD 1	1.14 ± 0.04	1.15 ± 0.03	0.76
POD 2	1.15 ± 0.06	1.13 ± 0.03	0.09
POD 3	1.17 ± 0.04	1.16 ± 0.03	0.70

Table 4 shows postoperative outcome variables. Among the postoperative variables, the prevalence of POAF and duration of ICU and hospital stay were statistically significant (P<0.05). The frequency of POAF in the elderly and young groups was 48.3% and 20%, respectively. The duration of ICU stay was 3.43 ± 1.61 days and 2.51 ± 1.30 days, respectively, in the elderly and young groups. The duration of hospital stay in the elderly and young groups was 10.03 ± 2.12 days and 8.88 ± 1.30 days, respectively.

**Table 4:** Postoperative outcome variables. Note: POAF (n = 29) in the elderly group and (n = 12) in the young group. POAF: postoperative atrial fibrillation; POD: postoperative day; ICU: intensive care unit

Variables	Elderly (n = 60) f (%)	Young (n = 60) f (%)	P-value
Hemoglobin (g/dL) in POD 1 (mean ± SD)	12.03 ± 1.33	12.10 ± 0.83	0.74
Plasma BNP (pmol/L) in POD 1 (mean ± SD)	198.31 ± 97.64	170.72 ± 68.07	0.07
POAF	29 (48.3%)	12 (20%)	0.002
Restoration to sinus rhythm from POAF (≤POD 7)	(n = 29), 23 (79.3%)	(n = 12), 10 (83.3%)	
Duration of ICU stay (days) (mean ± SD)	3.43 ± 1.61	2.51 ± 1.30	0.001
Duration of hospital stay (days) (mean ± SD)	10.03 ± 2.12	8.88 ± 1.30	0.001
Stroke	01 (1.7%)	0 (0%)	1.0
Mortality	0 (0%)	0 (0%)	

Table 5 shows the logistic regression analysis model of predictors of POAF following OPCAB. In an unadjusted logistic regression model, age (odds ratio = OR 3.74, 95% confidence interval (CI) = 1.66-8.41), preoperative plasma BNP (OR = 1.01, 95% CI = 1.00-1.01), and LA diameter (OR = 1.10, 95% CI = 1.03-1.17) were significantly associated with POAF (p < 0.05). However, in an adjusted logistic regression model, only age (OR = 1.31, 95% CI = 1.14-1.52, p < 0.0001) was significantly associated with POAF following OPCAB.

**Table 5:** Logistic regression analysis model of predictors of POAF following OPCAB. OR: odds ratio; CI: confidence interval; MI: myocardial infarction; BNP: B-type natriuretic peptide; LA: left atrial; LVIDD: left ventricular internal diameter at end-diastole; LVEF: left ventricular ejection fraction

Variable	Logistic regression analysis			
	Unadjusted model		Adjusted model	
	OR (95% CI)	P-value	OR (95% CI)	P-value
Age	3.74 (1.66-8.41)	0.001	1.31 (1.14-1.52)	<0.0001
Sex	0.86 (0.36-2.04)	0.73	0.33 (0.08-1.34)	0.12
Hypertension	0.78 (0.33-1.86)	0.58	1.52 (0.40-5.73)	0.53
History of old MI	0.26 (0.07-0.97)	0.045	0.35 (0.06-1.98)	0.23
Preoperative plasma BNP	1.01 (1.00-1.01)	0.001	1.00 (0.99-1.01)	0.17
LA diameter	1.10 (1.03-1.17)	0.001	1.06 (0.97-1.16)	0.17
LVIDD	1.03 (0.97-1.09)	0.27	0.93 (0.83-1.05)	0.26
LVEF	0.96 (0.91-1.01)	0.11	0.98 (0.88-1.09)	0.75
Left main stem involvement	0.55 (0.25-1.21)	0.14	0.74 (0.26-2.12)	0.58
Endarterectomy	0.30 (0.13-0.73)	0.008	0.31 (0.08-1.20)	0.09

## Discussion

This is the first study in Bangladesh that found ~48% of elderly off-pump CABG patients develop new-onset POAF. The findings are compatible with the results described in previous studies [1,7,16,19]. Advanced age was an independent predictor of AF following OPCAB. Earlier research has revealed patients of older age are more susceptible to POAF after CABG, but comparison with their younger counterparts is imprecise. This study in Bangladesh is the first to identify advancing age as the primary risk factor for POAF after OPCAB in contrast to young adults. Therefore, the rationality of this research indicates differences and variability with previous studies.

POAF significantly influences postoperative outcomes following CABG [12,16-20]. Moreover, POAF is not associated with BMI, diabetes mellitus, and COPD [21,22]. Although previous studies found smoking [10] and hypertension [22] to be significantly associated with POAF, we did not find any significant association with POAF among the Bangladeshi population. Unlike Tavakol et al. [23], we observed significantly higher mean plasma BNP levels in the preoperative period in both groups, which correlated to the findings of a previous study [11]. Albeit in an unadjusted regression model, plasma BNP is associated with POAF but in an adjusted regression model, not associated with POAF, which might be explained as other conditions are also responsible for the elevation of plasma BNP, which could not be excluded from the variable analyzed. We found comparably larger mean LA size in the elderly than in the young group, and LA size is the potential predictor of POAF following OPCAB. Furthermore, Xu et al. reported that increased LA size is associated with POAF, similar to our study findings [10].

Although left main stem disease [7] is significantly associated with POAF, previous studies reported that the number of diseased vessels [24] and right coronary artery occlusion [25] have no interdependence with the development of POAF, concordance with our findings. However, we observed a fairly high prevalence of left main stem involvement, which includes both significant (≥50% luminal stenosis) and non-significant (<50% luminal stenosis) left main disease, which might indicate the influence of the aging process on coronary arteries. Furthermore, we did not find any correlation between left main stem involvement and POAF.

In this study, the prevalence of in-stent restenosis was relatively high in the elderly than in the young, although a recent study reported an occurrence of about 3-20% [26]. Over two-thirds of the total endarterectomy was done in the left coronary territory. Our findings are consistent with earlier research [27]. Although coronary endarterectomy is associated with POAF [27,28], our study did not find any significant association between coronary endarterectomy and POAF. This study found no significant imbalance in mean serum electrolytes (Na^+^, K^+^, Ca^++^, and Mg^++^) level up to the third postoperative day and predicts that electrolyte imbalance might not be associated with POAF.

The prevalence of new-onset POAF was significantly higher in the elderly (48.3%) than in the young (20%), and the findings are similar to the observed frequency (20-40%) in recent studies [1,6,7]. We also found that the majority of the observed POAFs were paroxysmal AF. More than three-fourths of the POAF in both groups reverted spontaneously to normal sinus rhythm by conservative management using amiodarone and low-dose β-blocker, in which most of the POAF reverted to sinus rhythm within the first 24 hours. In a recent study, Lewicki et al. [22] reported that 78.6% of patients returned to sinus rhythm from POAF. Although POAF significantly prolongs the mean ICU stay [8,19,21,22] and hospital stay [8,19,21,22] among post-CABG patients, similar to the findings of this study. Prolonged ICU and hospital stay in the elderly indicates the influences of advancing age over the postoperative outcome after OPCAB.

This study has a few limitations, such as being conducted in a single center with a relatively small sample size, which may not represent the generalized population across Bangladesh and the world. In addition, this study followed the purposive sampling technique, and the lack of randomization during sampling may have contributed to the predictivity of POAF. Furthermore, the study sample also involved different surgeons with variations in techniques and preferences, which might result in a selection bias. Moreover, lack of resources such as flow probes to assess graft patency or epiaortic ultrasound to locate sites for proximal anastomosis to avoid atheroembolism, which might cause early graft failure resulting in POAF. Although this study has shown a high prevalence of POAF in elderly patients, a multicentered prospective randomized control trial with a large population in Bangladesh would reinforce the observations of this study.

## Conclusions

This study observed a comparably higher prevalence of POAF in the elderly following OPCAB in Bangladesh. Therefore, individuals with this complication needed more assistance during the hospital stay and at discharge. While advancing age independently predicts POAF in OPCAB patients, considering this, the stratification of patients with a higher risk of POAF should focus on reducing the occurrence of this common postoperative arrhythmia. Hence, our observations allow us to think about prophylactic approaches in the high-risk elderly population as the number of elderly considered for CABG is consistently increasing in Bangladesh.
